# Automated Retinal Vascular Analysis Reveals Response to Acetazolamide in Idiopathic Intracranial Hypertension

**DOI:** 10.1167/tvst.14.12.9

**Published:** 2025-12-04

**Authors:** Brian Woods, David Szanto, Jui-Kai Wang, Asala Erekat, Lola Stern, Aaron Golden, Mona K. Garvin, Randy H. Kardon, Mark J. Kupersmith

**Affiliations:** 1 Physics Department, School of Natural Sciences, University of Galway, Ireland; 2 Department of Ophthalmology, University Hospital Galway, Ireland; 3 Department of Ophthalmology, Icahn School of Medicine at Mount Sinai, New York, NY, USA; 4 New York Eye and Ear Infirmary of Mount Sinai, New York, NY, USA; 5 Department of Ophthalmology, University of Texas Southwestern Medical Center, Dallas, TX, USA; 6 Clinical Neuro-Informatics Program, Icahn School of Medicine at Mount Sinai, New York, NY, USA; 7 Department of Ophthalmology and Visual Sciences, University of Iowa Hospitals and Clinics, Iowa City, IA, USA; 8 Department of Electrical and Computer Engineering, University of Iowa, Iowa City, IA, USA; 9 Iowa City VA Center for the Prevention and Treatment of Visual Loss, Iowa City, IA, USA

**Keywords:** papilledema, idiopathic intracranial hypertension, retinal vascular analysis, automated image analysis, automorph

## Abstract

**Purpose:**

To assess whether automated analysis of retinal arterioles and venules can identify treatment response in papilledema secondary to idiopathic intracranial hypertension (IIH).

**Methods:**

This retrospective analysis used data from a multicenter, randomized, double-blind, placebo-controlled IIH treatment trial. Participants (*n* = 165) with mild visual loss were assigned to a dietary/lifestyle modification plus acetazolamide (ACZ) or placebo for 6 months. Color fundus photographs, optical coherence tomography (OCT), and clinical metrics were collected at baseline and at multiple follow-up visits. AutoMorph, a deep learning–based pipeline, quantified venule and arteriole diameters, fractal dimensionality, tortuosity, and vessel density. Venular widths were standardized to arteriolar widths to form a venule-to-arteriole (V:A) ratio, which was correlated with Frisén grade, OCT optic nerve head (ONH) parameters, and cerebrospinal fluid (CSF) opening pressure.

**Results:**

Baseline vascular OCT metrics and Frisén grades were similar between groups. At month 1, ACZ significantly reduced venule diameters (−4.59 µm; *P* = 0.02), and placebo showed no change (+1.21 µm; *P* = 0.54). The V:A ratio was consistently lower in the ACZ group than placebo from month 1 (1.20 vs. 1.24, respectively; *P* = 0.03) to month 6 (1.16 vs. 1.23, *P* = 0.02). Higher Frisén grades correlated strongly with increased mean V:A values (*R*^2^ = 0.91, *P* = 0.011). The V:A ratio was significantly associated with CSF opening pressure at month 6 (*R*^2^ = 0.47, *P* < 0.001).

**Conclusions:**

Automated retinal vessel analysis provides a promising, non-invasive method for monitoring treatment response in IIH and may complement traditional imaging and clinical assessments.

**Translational Relevance:**

Deep learning–based retinal vessel metrics may provide an accessible biomarker for monitoring treatment response in papilledema.

## Introduction

Idiopathic intracranial hypertension (IIH) is a disorder that principally affects young overweight women due to increased intracranial pressure (ICP) without an identifiable cause. It presents with headaches, pulsatile tinnitus, and binocular diplopia visual disturbances such as transient visual obscurations or actual vision loss due to papilledema.^1^ If left untreated, papilledema-related vision disturbances can progress to progressive and permanent vision loss.

Timely and accurate assessment of ICP and the degree of papilledema is vital in IIH management to prevent irreversible vision loss, monitor disease progression, alleviate symptoms, and guide treatment decisions. Weight management is crucial, and surgical options such as optic nerve sheath fenestration, ventricular peritoneal shunt, and venous stenting are used in specific situations.^2^^,^^3^ The Idiopathic Intracranial Hypertension Treatment Trial (IIHTT) was a multicenter, double-blind, randomized, placebo-controlled study that established that acetazolamide (ACZ), a carbonic anhydrase inhibitor, in addition to diet and lifestyle modifications, effectively reduces cerebrospinal fluid (CSF) pressure, improves visual outcomes, and reduces papilledema in patients with mild vision loss due to IIH.^4^^,^^5^ However, before considering invasive tests or therapies, accurate diagnosis and a reliable assessment plan to monitor treatment response are needed.^6^^,^^7^

Papilledema associated with elevated ICP develops because the increased pressure in the subarachnoid space is transmitted along the optic nerve sheath to the sclera and optic nerve head (ONH) in the retrolaminar region, resulting in ONH swelling. Although reducing ICP can improve clinical outcomes, the lag between ICP normalization and papilledema resolution complicates treatment response evaluation. This is further complicated by the growing deficiency in performing ophthalmoscopy of swollen optic discs and the use of an ordinal papilledema grading scale that may not reflect small changes.^7^ Even repeated lumbar punctures, which may spuriously show peaks or troughs, may not accurately reflect the pressure in the ONH and subarachnoid space in the retrobulbar region. Furthermore, distinguishing true papilledema from conditions such as optic disc drusen (pseudopapilledema) or other causes of ONH swelling remain challenging with conventional methods.^8^

Retinal venous changes are emerging as noninvasive indicators of elevated ICP.^9^ Moss et al.^10^^,^^11^ validated a semi-automated method for retinal vessel measurement using scanning laser ophthalmoscopy and showed that retinal venule diameter decreases with treatment, mirroring papilledema resolution. Ghate et al.^12^ confirmed in a pig model that acute ICP elevation causes reversible retinal venule dilation while Morgan et al.^13^ demonstrated that retinal venule pulsation correlates more closely with ICP than intraocular pressure, suggesting direct transmission of ICP to retinal veins. Zhang et al.^14^ further highlighted retinal imaging as a promising noninvasive ICP monitoring modality. Despite this potential, no widely accepted, non-invasive method currently exists to accurately reflect the pathological pressure increase at the ONH. Although papilledema grading, spectral-domain optical coherence tomography (OCT), and ultrasound are used in clinical practice, each has inherent limitations.^7^^,^^15^^–^^17^

Cessation of spontaneous venous pulsations (SVPs) is a well-established sign of raised ICP and retinal venule dilation; it has long been recognized heuristically in the clinical assessment of papilledema, but it lacks standardized assessment methods.^18^^,^^19^ The literature is filled with various approaches to measure the potential effects of venous hypertension in the retinal veins that occurs secondarily due to elevated ICP but none has been widely adopted. For example, ophthalmodynamometry has demonstrated strong correlations between ICP and retinal venous pressure, but its discomfort, operator dependency, and inability to monitor changes over time limit its utility.^20^^,^^21^

To overcome limitations in retinal vessel analysis, deep learning–based tools such as AutoMorph offer automated and objective assessments of retinal vascular morphology.^22^ Chen et al.^23^ recently demonstrated its utility in the assessment of patients with sickle cell disease. AutoMorph analyzes high-resolution fundus photographs to quantify vascular parameters, including venule and arteriole diameter. These metrics offer valuable insights into retinal vascular congestion and autoregulatory function, both of which are affected by changes in ICP. Automated analysis may enable earlier identification of treatment response compared to traditional methods. Additionally, the non-invasive nature and cost of retinal imaging make it ideal for frequent monitoring, improving patient compliance and reducing the need for invasive procedures such as lumbar puncture.

We hypothesized that patients receiving ACZ would demonstrate significant reduction in venule diameter compared to those receiving placebo prior to change in papilledema grade.^24^ We also hypothesized that an increase in venular diameter with raised ICP is due to an increase in central nervous system venous pressure, independent of papilledema severity, and may complement imaging of optic nerve edema. We sought to validate automated retinal vessel analysis as a non-invasive tool for monitoring disease progression and treatment efficacy in IIH. Additionally, we aimed to determine the relationship between the change in the volume of optic disc derived from OCT and change in retinal vasculature metrics.^25^ We also explored whether venular width corresponds to CSF opening pressure prior to treatment and after treatment.

## Methods

### Study Design and Participants

The Institutional Review Board (IRB) of the Icahn School of Medicine at Mount Sinai waived the need for additional approval or consent, as the data were de-identified and obtained from participants who had previously provided consent, and multiple site IRBs had approved use of the clinical trial data for research purposes. The study adhered to the tenets of the Declaration of Helsinki.

### Study Participants

We acquired IIH fundus photographs from the IIHTT (NCT01003639), a National Institutes of Health–sponsored multicenter, randomized, double-blind, placebo-controlled trial for which the full details and additional findings have been previously published.^4^ In brief, 165 individuals with IIH and mild vision loss (defined as a perimetric mean deviation between −2 dB and −7 dB) were enrolled at 38 centers across North America between 2010 and 2012. All participants were provided with a dietary plan and lifestyle modification program, and they were randomized to receive either maximally tolerated ACZ (up to 4 g/d) or a placebo for a period of 6 months.^26^

### Eyes Analyzed

The eyes included in this analysis were the designated study eyes (one per subject) identified at trial entry. We validated the findings in a separate cohort comprised of fellow eyes of the same participants. Color fundus photographs encompassed optic disc grades assigned by experts as Frisén grades 0 through 5. A total of 4102 images were collected across multiple study visits from initial baseline visit to month 6. We additionally analyzed 2932 fundus photos of healthy eyes taken from locally-sourced high-resolution fundus photos, grade 0 ‘Diabetic Retinopathy Disease dataset’ on Kaggle, and ‘1000 Fundus images’ on Kaggle.^27^^,^^28^

### Image Processing and Analysis

**Figure 1.**
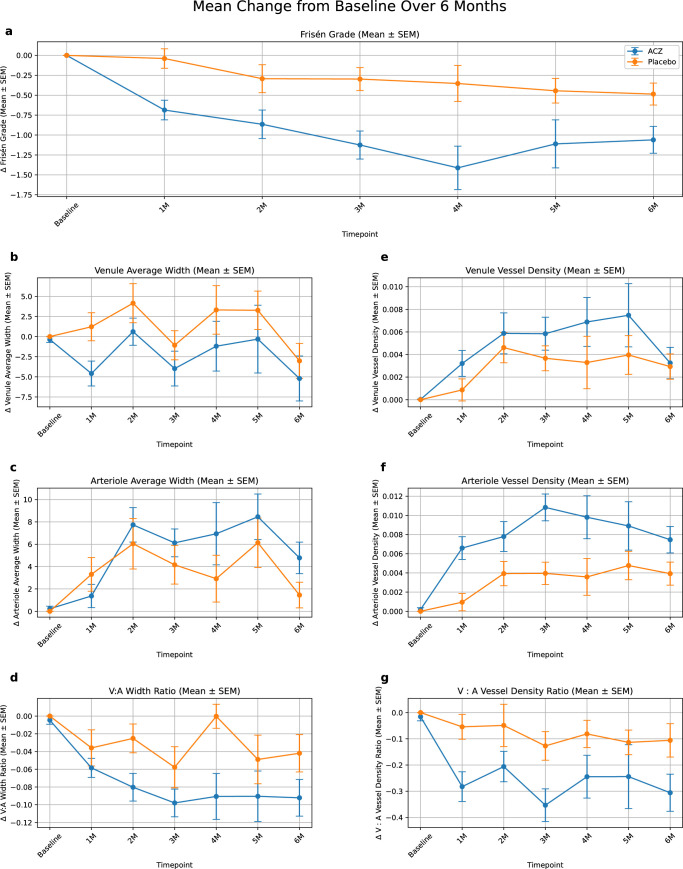
(**a–g**) Mean ± SEM changes over 6 months in Frisén grade (**a**), venule average width (**b**), arteriole average width (**c**), V:A ratio (**d**), venule vessel density (**e**), arteriole vessel density (**f**), and V:Avd ratio (**g**). Frisén grade improved in ACZ group compared with the placebo group (−1.12 vs. −0.49, respectively; *P* < 0.001) by month 6 (**a**). When venular measurements (**b**) were standardized using their corresponding arteriolar measurement (**c**), the ACZ group saw a greater decrease in mean V:A ratio versus placebo (**d**) over the course of 6 months (−0.10 vs. −0.04, respectively; *P* < 0.001). Likewise, when venule vessel density measurements (**e**) were standardized using their corresponding arteriole vessel density measurements (**f**), the ACZ group saw a more pronounced reduction in standardized V:A vessel density metrics (**g**).

We utilized the AutoMorph pipeline to conduct the automated analysis of fundus photographs as this is a high-quality segmentation tool previously shown to have an intervisit minimum segmentation accuracy of 97%.^29^ Briefly, this pipeline involves several key steps (see Fig. 1):

1. Utilizing the EyeQ automatic quality control system developed by Fu et al.,^30^ we adjusted the cut-off threshold to 0.90, as these images came from a clinical trial where fundus photographs were a primary method of disease assessment and hence were of high quality. By increasing this threshold, we reduced the chance of images being rejected due to ONH swelling.
2. The pipeline then identified the ONH to define the analysis region (lwnet).^31^
3. Retinal vessels were classified as either arterioles or venules using BF-Net, a GAN-based segmentation backbone.^32^
4. Vessel morphology was assessed across various domains, and average vessel width measurements were extracted using Retipy and a pixel scaling factor of 0.008 mm/pixel, consistent with the typical scale of a 35° to 45° fundus image.^33^ This represents an approximate physical scale; therefore, to minimize dependence on absolute calibration, we standardized venular metrics relative to arteriolar values, deriving the venule-to-arteriole width (V:A) ratio and venule-to-arteriole vessel density (V:Avd) ratio as the main vascular endpoints.

For each patient, we had multiple photographs at each time point and thus took the mean of each metric at each time point.

### OCT Data

A subset of 126 IIHTT participants underwent CIRRUS spectral-domain, ONH-centered OCT volumes covering 6 × 6 × 2 mm^3^ (Zeiss-Meditec, Inc, Dublin, CA) as part of the IIHTT OCT sub-study at baseline and at 6 months.^34^ The peripapillary ONH volume (pONHV) was defined as the volume of tissue between the internal limiting membrane and the lower boundary of the retinal pigment epithelium complex, within a 1.73-mm-radius circle centered at the optic disc. The peripapillary total retinal thickness (pTRT) and peripapillary retinal nerve fiber layer (pRNFL) thicknesses were also analyzed.

### Lumbar Puncture Opening Pressure Data

CSF opening pressures, obtained using the study protocol method, at baseline (*n* = 155) and V:A ratio change data between baseline and at month 6 were available for 50 participants.

### Statistical Analyses

We performed all statistical analyses using Python 3.9.12. Within-group changes in retinal measurements (i.e., baseline vs. follow-up time points) were assessed using paired *t*-tests at an alpha of 0.05. Between-group comparisons of change (i.e., ACZ vs. placebo) were evaluated using independent *t*-tests. *P* values were adjusted for multiple comparisons using the Benjamini–Hochberg false discovery rate correction. Association with Frisén grade was reported with ANOVA testing. Tukey's post hoc testing with multiple comparison of means was performed for V:A ratio across all Frisén grades. We used Pearson correlation to quantify the linear relationship between Frisén grade (treated as a numeric ordinal variable) and the group-level mean V:A ratio. Pearson correlations were also used to assess the relationship between V:A ratio and CSF opening pressure. We used multiple linear regression to assess the relationship between change in V:A ratio (average of both eyes) and CSF opening pressure change at 6 months. For each time point, values across both eyes were averaged per patient to reduce inter-eye variability.

To assess treatment effects across all visits in a unified framework, we implemented a linear mixed-effects model including fixed effects for time, treatment, and their interaction, with random intercepts for subjects to account for repeated measures. The treatment × time interaction term captured the differential rate of change between ACZ and placebo groups. All continuous outcome variables were standardized (*z*-scored) prior to modeling to facilitate comparison of effect sizes across outcomes.

## Results

### Quality Assessment and Data Inclusion

Automatic quality control excluded 199 photographs across treatment arms and visits (Supplementary Table S2). The mean Frisén grade of excluded images was 2.75. We analyzed 5224 optic disc–centered fundus photographs from 165 participants enrolled in the IIHTT from baseline to month 6. There were 2556 study eye fundus photographs from 155 eyes that passed AutoMorph quality assessment (80 ACZ, 75 placebo). After aggregating multiple scans of the same time point, we had 690 time points for analysis. We utilized the fellow eyes for a separate validated analysis (80 ACZ, 72 placebo). We additionally analyzed 2932 normal eyes.

### Changes in Frisén Grade

Frisén grades were similar for the ACZ and placebo groups at baseline (2.80 vs. 2.84, respectively; *P* = 0.81). There was a difference in mean Frisén grade at month 1 between the ACZ and placebo groups (2.19 vs. 2.67, respectively; *P* < 0.05). The Frisén grade was reduced in the ACZ group compared with the placebo group at 6 months (mean change from baseline, −1.12 vs. −0.48, respectively; *P* < 0.01) (Fig. 1a, Supplementary Table S1). In fellow eyes, Frisén grades decreased significantly in the ACZ group from baseline to month 6 (mean change from baseline, −1.14; *P* < 0.001), mirroring findings from study eyes. Placebo-treated fellow eyes also showed a significant but more modest decline (mean change from baseline, −0.34; *P* = 0.048).

### Changes in Venule Diameter

At baseline, the mean venule diameters for the ACZ and placebo groups were 140.9 µm and 137.8 µm, respectively (*P* = 0.93). At 1 month, the ACZ group showed a greater reduction in venule width compared with the placebo group (−4.59 µm vs. +1.22 µm, respectively; *P* = 0.014). At 6 months, the ACZ group showed a statistically significant reduction in venule average width (−6.28 µm; *P* = 0.039) from baseline, but the placebo group did not (−3.09 µm; *P* = 0.20). The difference between the two groups in terms of their change in venule width from baseline was not statistically significant at any time point beyond 1 month (Fig. 1b, Supplementary Table S1). Venular width also decreased over time in the ACZ-treated fellow eyes (−10.07 µm at month 6; *P* = 0.003).

### Changes in Arteriole Diameter

The arteriolar measurements were similar at baseline for ACZ and placebo groups (112.7 µm vs. 110.3 µm, respectively; *P* = 0.52). Both groups showed significant increases in arteriolar width: ACZ from month 2 onwards (+7.89 µm, *P* < 0.001); placebo group from month 1 (+3.30 µm, *P* = 0.046). At 6 months, ACZ showed a +4.35 µm (*P* = 0.01) increase from baseline, whereas the placebo group showed a 1.26 µm increase that was not statistically significant (*P* = 0.21). The difference between the two groups in terms of their change from baseline was not statistically significant at any time point (see Fig. 1c, Supplementary Table S1). Similarly, a consistent and significant increase in arteriolar width was observed in fellow eyes treated with ACZ. From baseline to month 6, the mean change in arteriolar width was +4.48 µm (*P* = 0.056), with the largest increase from baseline observed at month 4 (+10.06 µm; *P* = 0.001). This pattern closely mirrored that of the study eyes.

### Changes in Vessel Density

Measurements of baseline overall vessel density (i.e., the ratio between the area of all vessels, including arterioles and venules, to the whole image) for ACZ or placebo groups were similar (0.07 vs. 0.07, respectively; *P* = 0.95). The ACZ group showed a significant increase in overall vessel density from month 1 onward (+0.007; *P* < 0.001), whereas this increase did not become apparent in the placebo group until month 2 (+0.006; *P* < 0.01). Looking specifically at venule vessel density, we saw increased venule density from month 1 in the ACZ group (+0.003; *P* = 0.013) and from month 2 in the placebo group (+0.005; *P* = 0.003), but the difference between groups was not significant at any time point. In contrast, arteriole vessel density demonstrated a significant difference between the ACZ and placebo groups at month 1 (+0.007 vs. +0.001, respectively; *P* = 0.009). A significant difference between the ACZ and placebo groups was noted from month 1 onward when venule vessel density was standardized by the V:Avd ratio (−0.27 vs. −0.05, respectively; *P* < 0.001) (Fig. 1g). In fellow eyes, total vessel density increased by an average of +0.010 units in the ACZ group versus +0.003 in the placebo group (*P* = 0.044) over the course of the study. Arteriole vessel density also showed a between-group difference at month 6 for the ACZ and placebo groups (0.036 vs. 0.031, respectively; *P* = 0.029). The fellow eye V:Avd ratios were similar between both ACZ and placebo at baseline (1.46 vs. 1.50, respectively; *P* = 0.62) and showed a significant difference between the two groups by month 1 (1.27 vs. 1.48, *P* < 0.001).

### Changes in Tortuosity Density

Tortuosity density measurements for the ACZ and placebo groups at presentation were similar (0.71 vs. 0.71, respectively; *P* = 0.49). AutoMorph did not identify any significant change in tortuosity density out to 6 months for either group (0.714 vs. 0.715, respectively; *P* = 0.921). A significant reduction in venule tortuosity density from baseline was noted at 1 month in the ACZ group (*t*-statistic = −2.43; *P* = 0.019) but not in the placebo group (*t*-statistic = −0.13; *P* = 0.89), and no significant difference was noted between the groups. No significant change in artery tortuosity density was noted at any stage in either group. Fellow eyes did not reveal any significant differences or changes between groups for tortuosity density.

### Changes in Distance Tortuosity

ACZ and placebo groups displayed similar retinal vessel distance tortuosity at baseline (4.01 vs. 3.67, respectively; *P* = 0.11). There was no significant change in overall distance tortuosity between the groups over 6 months (4.19 vs. 3.82; *P* = 0.28). No significant changes were noted in venule distance tortuosity over the 6 months. Arteriole distance tortuosity showed an increase from baseline in the ACZ group at 1 month (+0.81; *P* < 0.001), whereas there was no significant change from baseline in the placebo group at any stage. No notable differences or changes were observed in the fellow eyes.

### Changes in Squared Curvature Tortuosity

There was no difference in baseline squared curvature tortuosity for the ACZ or placebo groups (39.4 vs. 30.7, respectively; *P* = 0.11). There was no significant difference between groups over 6 months (47.3 vs. 53.3, respectively; *P* = 0.23). Looking specifically at venule squared curvature tortuosity, there was a significant difference between the ACZ and placebo groups at 6 months (30.0 vs. 53.4, respectively; *P* = 0.039). Analyses of fellow eyes revealed no meaningful change or between-group variation.

### Changes in Fractal Dimensionality

Baseline fractal dimensionality was similar for the ACZ and placebo groups (1.45 vs. 1.44, respectively; *P* = 0.45). At month 1, the ACZ group showed a greater increase in fractal dimensionality than the placebo group (+0.032 vs. −0.002, respectively; *P* < 0.001). Looking specifically at venule fractal dimensionality, ACZ increased relative to placebo at month 1 (+0.030 vs. +0.004, respectively; *P* = 0.018), but there was no difference between treatment groups at any other time point. The ACZ group demonstrated increased arteriolar fractal dimensionality compared with placebo at month 1 (+0.057 vs. +0.010, respectively; *P* = 0.002). Fractal dimensionality metrics increased significantly in fellow eyes of the ACZ group. ACZ showed a greater change in total fractal dimensionality from month 1 onward (+0.020 vs. 0.000, respectively; *P* = 0.007). Venule fractal dimensionality showed a greater change from baseline for ACZ than placebo from month 3 (+0.046 vs. +0.014, respectively; *P* = 0.013), and arteriole fractal dimensionality demonstrated a difference from month 1 (+0.044 vs. +0.006, *P* = 0.045).

### Changes in Venule-to-Arteriole Ratios

At baseline, mean V:A ratio measurements for the ACZ and placebo groups were similar (1.25 vs. 1.26, respectively; *P* = 0.83 (see Supplementary Material)). The ACZ group showed a reduced V:A ratio compared to the placebo group at all time points from month 1 (1.20 vs. 1.24, respectively; *P* = 0.034) to month 6 (1.16 vs. 1.23, *P* = 0.02) aside from month 5 (1.17 vs. 1.19, *P* = 0.45) due to a lack of data at this time point (not one of the primary data collection points of the study) (Fig. 1d, Supplementary Table S1). By 6 months, the ACZ group showed a −0.10 reduction in V:A ratio (*P* < 0.001) from baseline. In contrast, the placebo group did not show a significant change (−0.04; *P* = 0.07). In fellow eyes, the V:A ratio also declined steadily in the ACZ group, reaching statistical significance by month 1 and persisting through month 6 (−0.14; *P* < 0.001). Placebo-treated fellow eyes showed minimal change in V:A ratio over the 6 months (−0.01; *P* = 0.68).

Repeatability analysis of study eye V:A width ratio measurements from separate fundus photographs acquired at the same time point (*n* = 566) demonstrated an intraclass correlation coefficient of 0.84 (95% confidence interval [CI], 0.81–0.87), indicating good within-eye reliability across two repeated measurements. Averaging a greater number of V:A ratio measurements from repeated fundus photographs at each time point further improved measurement consistency (see Supplementary Fig. S2).

### Association of V:A Ratio with Frisén Grade

**Figure 2.**
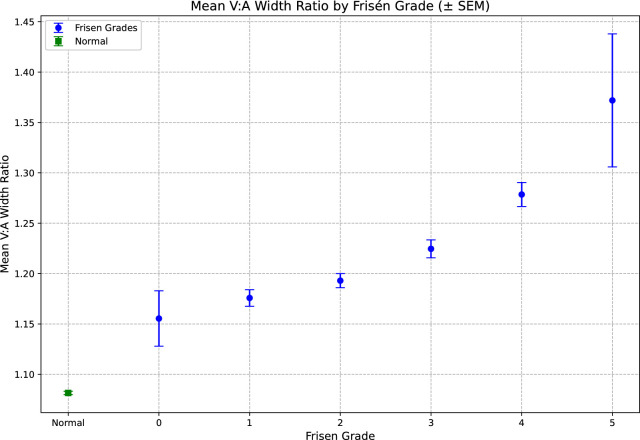
Increasing Frisén grades are associated with increased V:A ratio. There was a significant overall difference in V:A ratios across Frisén grades (ANOVA *P* < 0.0001). Post hoc analysis revealed that the difference between grades 1 and 2 was not significant. However, moving from grade 1 to grades 3, 4, and 5 showed significant increases in V:A ratio. Likewise, all other pairwise comparisons were significant, indicating that higher Frisén grades tend to be associated with higher V:A ratio values. There was a significant difference between normal eyes and eyes that had papilledema (*P* < 0.0001).

**Figure 3.**
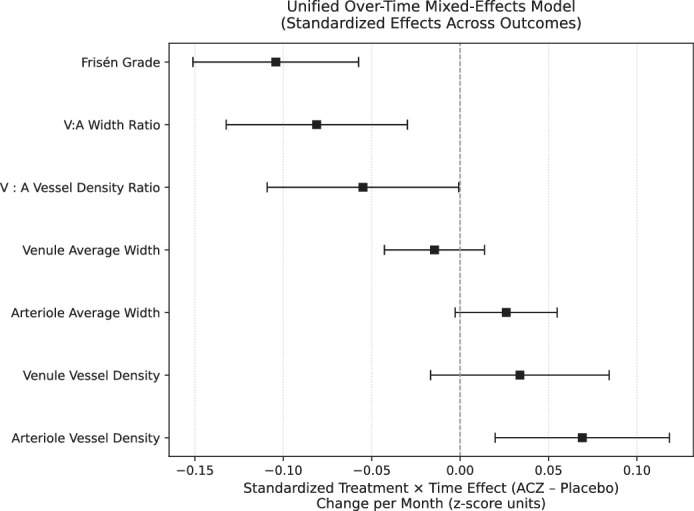
Forest plot of standardized over-time treatment effects (ACZ/placebo) from unified mixed-effects models. Each row represents a vascular or structural outcome, showing the difference in rate of change between ACZ and placebo across all study visits. Estimates are expressed as standardized change per month (*z*-score units), allowing comparison across outcomes with different measurement scales. Negative values indicate greater reduction (or faster improvement) with ACZ relative to placebo, whereas positive values indicate relative increases with ACZ.

ANOVA testing revealed a significant difference in V:A ratios among the different Frisén grades (*P* < 0.001). Across all time points, the mean ± SD V:A ratio for Frisén grades 1, 2, 3, 4, and 5, respectively, were 1.17 ± 0.1, 1.19 ± 0.1, 1.22 ± 0.1, 1.27 ± 0.12, and 1.37 ± 0.21. Tukey's post hoc testing with multiple comparisons of means revealed significant differences in V:A ratios across all Frisén grades, except between grades 1 and 2. Higher Frisén grades were associated with increased mean V:A ratios (*R*^2^ = 0.91, *P* = 0.011) (Fig. 2). In the subset of 38 patients who did not have a change in Frisén grade by month 1 but went on to have a change in Frisén grade at a later time point, 10 demonstrated a change in V:A ratio (at a threshold of 0.01) before a change in Frisén grade, 27 had a V:A ratio change at the same time point as a change in Frisén grade, and one demonstrated a change in V:A ratio at a later time point than a change in Frisén grade. In all cases the change in V:A ratio was in the same direction as the change in Frisén grade. Results from the linear mixed-effects model (Fig. 3, Supplementary Table S3) demonstrated significant over-time treatment effects for Frisén grade (−0.10; 95% CI, −0.15 to −0.06; *P* < 0.001), V:A ratio (−0.08; 95% CI, −0.13 to −0.03; *P* = 0.001), and V:Avd ratio (−0.06; 95% CI, −0.11 to 0.00; *P* = 0.046).

### Association of V:A Ratio With ONH Volume

**Figure 4.**
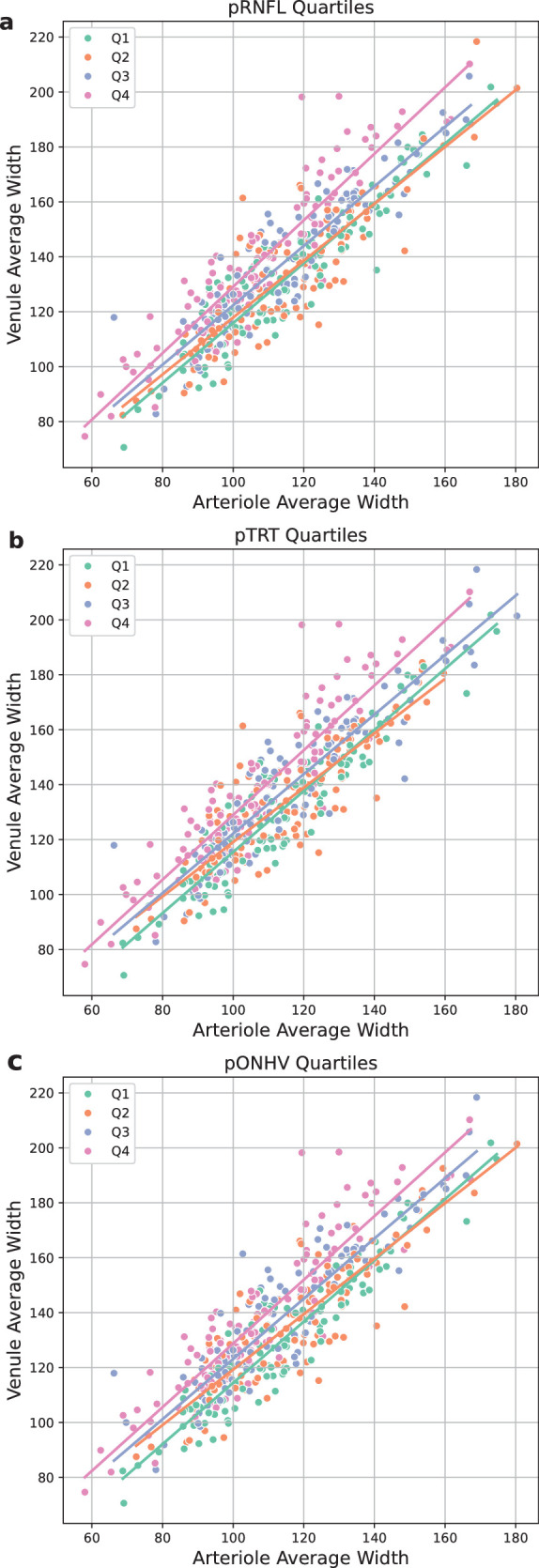
Average arteriolar width versus average venular width color-coded by OCT quartile measurement. (**a**) pRNFL thickness. (**b**) pTRT thickness. (**c**) pONHV. With increasing OCT metrics, we can see that the line representing V:A ratio steepens, indicating that average venular width was responsible for the increase in the V:A ratio.

From baseline to month 6, the ACZ group saw a greater decrease in pRNFL compared with the placebo group (−161 µm vs. −55 µm, respectively; *P* < 0.001), as well as in pTRT (−198 µm vs. −70 µm, *P* < 0.001) and pONHV (−4.4 mm^3^ vs. −1.44 mm^3^, *P* < 0.001). We found that, as OCT measurements of optic disc edema increased, venular width changes became more pronounced for each unit change in arteriolar width (*P* < 0.001) (Fig. 4). We observed a significant difference in V:A ratio across pRNFL (*P* < 0.001), pTRT (*P* < 0.001), and pONHV quartile groups (*P* < 0.001). Furthermore, a positive linear trend in V:A ratio was detected for pRNFL (β = 0.046, *P* < 0.001), pTRT (β = 0.045, *P* < 0.001), and pONHV (β = 0.048, *P* < 0.001), indicating that higher OCT ONH metrics were associated with proportionally wider venules relative to arterioles. Using a threshold of half a standard deviation to define a “meaningful change” in each OCT metric, we found that, in the majority of cases, the V:A ratio changed in the same direction as the OCT metric (concordance: pTRT, 89%; pRNFL, 88%; pONHV, 88%). Moreover, these changes typically occurred at the same time point for both modalities (83% for pTRT, 86% for pRNFL, and 82% for pONHV). Notably, the V:A ratio and OCT metrics changing in opposite directions occurred exclusively in placebo-treated patients.

### Association With Change in Lumbar Puncture CSF Opening Pressure

**Figure 5.**
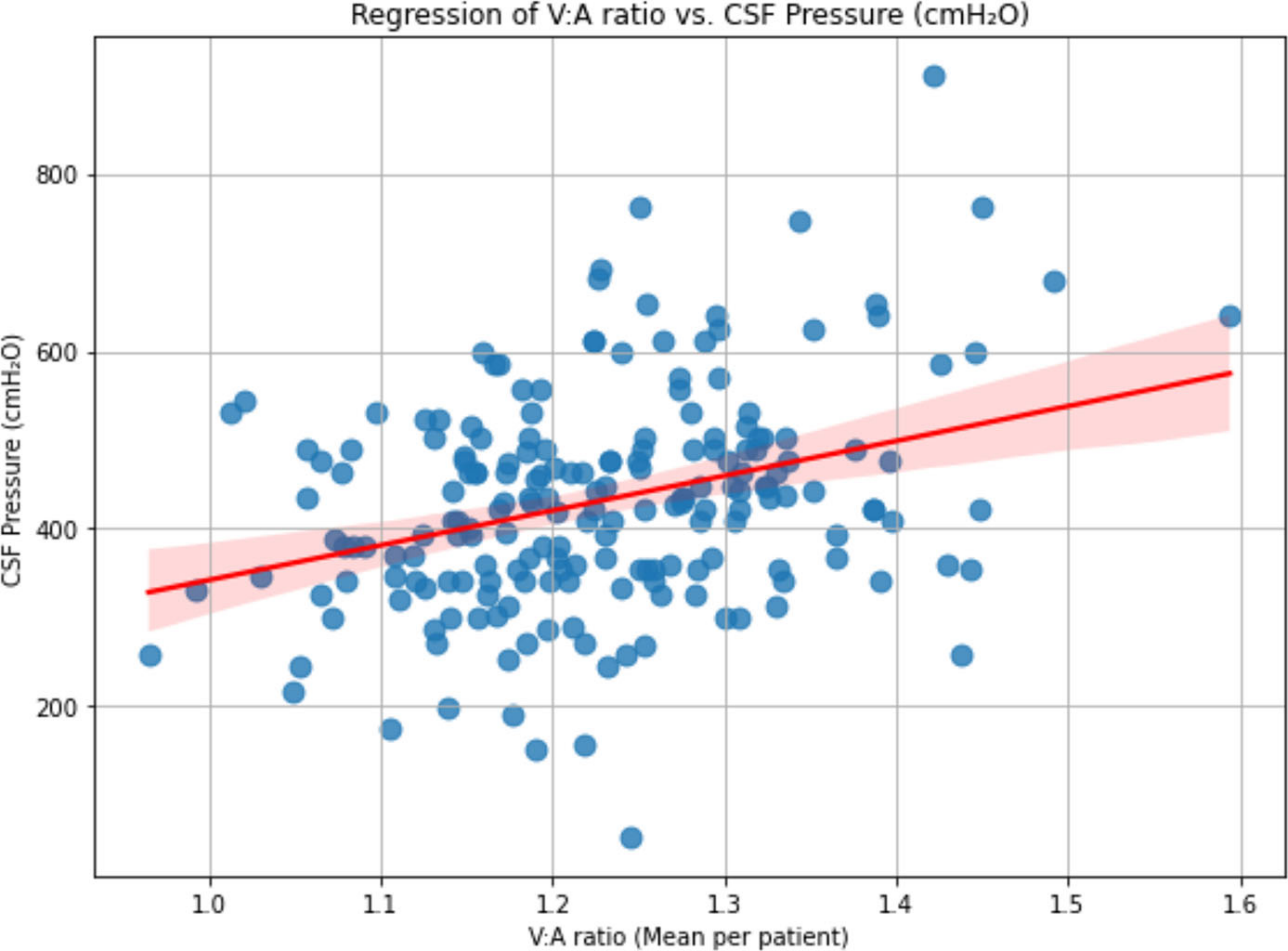
Correlation of mean study eye and fellow eye V:A ratio with lumbar puncture CSF opening pressure, where *r* = 0.33 and regression slope estimate = 391.8 (95% CI, 233.7–549.8; *P* < 0.001).

Both Frisén grade and V:A ratio correlated with lumbar puncture CSF opening pressure. For Frisén grade, *r* = 0.39 and regression slope estimate = 33.9 (95% CI, 22.5–45.1; *P* < 0.001). For V:A ratio, *r* = 0.33 and regression slope estimate = 288.1 (95% CI, 171.9–404.3; *P* < 0.001) (Fig. 5). Both the change in Frisén grade and change in V:A ratio correlated, respectively, with change in CSF opening pressure. For Frisén grade, *r* = 0.28 and regression slope estimate = 31.7 (95% CI, 1.19–62.1; *P* = 0.04). For V:A ratio, *r* = 0.35 and regression slope estimate = 302.9 (95% CI, 77.0–528.9; *P* < 0.01). A linear regression model incorporating baseline CSF opening pressure and the mean inter-eye change in V:A ratio from baseline to month 6 explained 47% of the variance in final CSF opening pressure (*R*^2^ = 0.47, *P* < 0.001; mean absolute error [MAE] = 80.8 cm H_2_O).

## Discussion

This study demonstrated that automated retinal vessel analysis using the AutoMorph platform can detect and quantify abnormal retinal vessels in papilledema. Notably, we observed that the V:A ratio and V:Avd ratio significantly decreased within 1 month of initiating treatment with ACZ. The significant reduction in venule diameter and changes in the V:A ratio observed in the ACZ group align with the previous finding of improved papilledema in the IIHTT.^4^^,^^11^ Validation in fellow eyes reinforced the reproducibility of our findings. Interestingly, these vascular changes preceded improvements in Frisén grade in some eyes, highlighting the potential of vascular analysis as a sensitive, early indicator of treatment response.

### Role of Automated Vascular Assessment

Traditional methods for evaluating ICP and papilledema have inherent limitations: Lumbar puncture is invasive and unsuitable for frequent monitoring, and fundoscopic examination (including ordinal, non-continuous grading systems such as the Frisén scale) is subject to inter-observer variability, does not change quickly, and may not reliably detect subtle changes in optic nerve swelling.^7^^,^^15^^,^^16^ Furthermore, the subjectivity of the clinical evaluation of features in the peripapillary retina and ONH results in significant variability between and within observers, and necessitates specialized clinical expertise. Ultrasonographic measurement of the optic disc requires a cooperative patient and is technician dependent. Spectral-domain OCT results can be marred by artifacts due to poor light transmission in markedly swollen ONHs. Work from our group has previously developed an automated segmentation algorithm to overcome some of the OCT machine algorithm failures due to acquired ONH swelling.^35^ However, OCT measurements of pRNFL thickness can still be confounded by chronic papilledema or optic atrophy, further complicating disease monitoring.

Emerging non-invasive techniques offer alternative solutions to these challenges. Recently, we utilized deep learning on fundus photographs to quantify the degree of papilledema and changes over time in response to ACZ treatment.^36^ Retinal venules are susceptible to congestion in the setting of elevated ICP, making their diameter a useful indicator of ICP dynamics.^11^^,^^20^

The absence of SVPs is a well-recognized indicator of elevated ICP, and dilation of the retinal venules has long been observed clinically in cases of papilledema.^18^ Monitoring these changes may be a potential avenue to non-invasively assess disease progression and evaluate the effectiveness of therapeutic interventions. Prior research has demonstrated a correlation between pressure in the central retinal vein as measured by ophthalmodynamometry and elevated ICP.^20^ Previous studies have reported retinal vascular changes in IIH, and our findings further support the use of retinal vascular analysis as an objective biomarker to complement existing methods for assessing disease severity and progression.^9^^–^^14^^,^^37^

Moss et al.^11^ employed standardized methods to assess the area of ONH elevation and measure the diameters of four arterioles and four venules in fundus photographs of patients with IIH. They showed how retinal vessel diameter changes can reflect treatment efficacy. However, manual annotations of retinal vessels and measurements are time consuming and prone to human error, whereas automated vascular analysis provides a more complete, objective, and global measure of vascular analysis in papilledema.

The use of the AutoMorph platform enhances the strength and clinical utility of our methodology by incorporating explainable artificial intelligence. Unlike black-box models, AutoMorph provides interpretable outputs based on well-defined anatomical and physiological parameters, such as venule and arteriole diameter, tortuosity, and fractal dimensionality.^22^ The standardization of this method of assessment overcomes the subjectivity and variability inherent in traditional methods such as Frisén grading.

### Vascular Findings

The reduction in venous diameter observed in the ACZ group, but not in the placebo group, is likely due to the lowering of ICP and, consequently, CNS venous pressure. Elevated ICP impairs venous outflow from the retina by increasing pressure within the central retinal vein and other intracranial venous channels, leading to venous dilation. Although optic disc edema may contribute locally to venous stasis by compressing the central retinal vein within the optic nerve sheath, our findings, particularly the early venous changes preceding improvements in Frisén grade, suggest that systemic CNS venous pressure is the primary driver of the venular dilation in the setting of IIH. Therefore, the observed decrease in venular width with ACZ treatment likely reflects the resolution of this upstream pressure rather than the resolution of local disc edema alone.

These findings are further supported by prior work examining Bruch's membrane displacement in IIH.^38^ In that study, displacement decreased in the ACZ group but not in the placebo group, despite both groups showing improvement in disc edema. This was interpreted as evidence that ICP may have remained elevated in the placebo group, with improvements in axoplasmic flow possibly reflecting compensatory mechanisms rather than true ICP normalization. Similarly, in our study, the reduction in venular diameter observed only in the ACZ group suggests that these vascular changes are more closely tied to reductions in CNS venous pressure than to local structural changes from optic disc edema alone. Supporting this interpretation, we found that the V:A ratio and OCT-derived edema metrics changed in the same direction and at the same time point in the majority of cases; however, when changes in V:A ratio and OCT metrics diverged, this occurred exclusively in placebo-treated patients. This suggests that venular metrics may more directly reflect underlying ICP dynamics, whereas OCT measures can be influenced by compensatory or biomechanical adaptations in the ONH.

In contrast to previous findings, our study noted an increase in arteriolar diameter in the ACZ group from 2 months. Mittra et al.^39^ previously reported increased blood flow in the central retinal artery and ONH following optic nerve decompression. Thus, our results may represent some element of improved autoregulatory function and reduced resistance in the ONH, improving central retinal artery flow as ICP decreases.

As expected, V:A ratio changes appear to be predominantly driven by increasing venular width (Fig. 4). As arterioles are more muscular structures, they appear to be more resistant to external fluctuations in pressure than the more compressible venules. Our study benefits from standardizing venular diameter relative to arteriole width within the same image, which minimizes the confounding effects of inter-photo magnification and field-of-view variability. This normalization enhances the reliability and comparability of vascular metrics across time points and patients, revealing features more closely aligned with Frisén grade. This reinforces the clinical relevance of vessel analysis as a sensitive and objective indicator of papilledema severity. The significant improvements in retinal vascular parameters and papilledema severity in the ACZ-treated group in our study demonstrate the potential for retinal vascular analysis, in particular the V:A ratio reduction, as a non-invasive, objective tool for monitoring IIH progression and treatment.

### V:A Ratio and Its Association With Papilledema Severity

Recognizing higher Frisén grades is important, as they are associated with higher risk of vision loss. Vascular analysis may capture data in a more granular, continuous fashion than is possible with the ordinal Frisén grade system. Our study shows that the V:A ratio is increased across higher Frisén grades. Importantly, Tukey's post hoc analysis revealed that higher Frisén grades were associated with elevated V:A ratios, further underscoring the relationship between retinal vascular changes and papilledema severity. Interestingly, on subset analysis, changes in the V:A ratio tended to occur before changes in Frisén grade were noted. Additionally, there was a significant difference in V:A ratio between fundus photographs of normal eyes and eyes with papilledema (see Fig. 1). The clear difference between normative data and IIH V:A ratios, even at low Frisén grades, supports the idea that non-local factors affect the V:A ratio besides compression within the disc at the central retinal vein. Venular response to elevated ICP may follow a biphasic pattern with initial V:A ratio increases due to passive venular dilation secondary to elevated transmural pressures. As ICP remains elevated, CSF pressure in the optic nerve sheath impairs central retinal vein drainage further, exacerbating dilation. Factoring in ONH biomechanical factors will be important to elucidate this mechanism further. In particular, it would be important to establish whether those with smaller ONH compartments are at increased risk of subsequent visual deficits.

### Vascular Tortuosity and Density Metrics

Our results are concordant with previous work that has shown that increases in venous pressure usually cause increases in venular tortuosity but not arteriolar tortuosity.^40^^,^^41^ Kylstra et al.^41^ previously found that vessel diameter and tortuosity respond differently depending on the severity of transmural pressure. Cao et al.,^42^ in their analysis of the vascular complexity of IIH patients using OCT angiography, reported reduced arteriole complexity and increased venule complexity in patients with IIH-associated papilledema versus control eyes. Interestingly, although venular and arteriolar vessel density measurements both demonstrated increases over time when we standardized venular measurements by their arteriolar measurements (V:Avd ratio), a clear recapitulation of the pattern of swelling reduction as measured by expert graders was seen for both placebo and ACZ. For ACZ, the rate of increase of arteriolar width was greater than that of venular width. This may point to a reversal of pressure-induced vascular compression and improved perfusion of the ONH. Although the relief of venous congestion may account for changes observed in venous fractal dimensionality, the increased fractal dimensionality of arterioles following ACZ treatment may possibly reflect restoration of vascular morphology and autoregulatory capacity. Of note, however, vessel density and fractal dimensionality metrics using the AutoMorph pipeline are subject to higher inter-photograph variability than other metrics, so results should be interpreted with caution.^29^

### Association With Change in Lumbar Puncture CSF Opening Pressure

Most interestingly, the V:A ratio correlated with lumbar puncture CSF opening pressure, and changes in the V:A ratio correlated with changes in CSF opening pressure. If further developed, this could be incorporated into a low-cost, non-invasive, and continuous predictive algorithm. Change in V:A ratio demonstrated a better correlation with CSF opening pressure than change in Frisén grade over the course of 6 months. By incorporating opening CSF pressure at baseline alongside V:A changes between baseline and month 6, our multiple linear regression model demonstrated a moderately strong correlation with CSF opening pressure at follow-up (*r* = 0.69, *P* < 0.001). Our data echo the findings reported by Hagen et al.,^37^ who observed similar trends in a smaller cohort. Their study similarly noted a measurable relationship between standardized vascular metrics and lumbar pressure across healthy and borderline individuals. This alignment across studies supports the further exploration of retinal vascular analysis as a potentially safe, less expensive, non-invasive methodology for ongoing monitoring of elevated ICP through incorporation with complementary traditional methods such as Frisén grading, visual field testing, and ONH measurements to offer a comprehensive approach to IIH management.

### Limitations

Several limitations should be acknowledged. Although data from specific clinical trials and observational cohorts provide valuable standardization in disease characterization, they may restrict the applicability of the findings to wider populations. The exclusion criteria for the IIHTT meant that patients with more severe disease were not included in this analysis.^26^^,^^43^ This meant that we had relatively fewer instances of grade 4 and 5 swelling and only patients with mild visual field defects. It is possible that AutoMorph may have lower accuracies of vessel segmentation and arteriole or venule identification in cases of severe swelling. Examples of vessel misclassifications can be seen in the Supplementary Material. Our results show pronounced changes in vascular metrics in higher grades of papilledema; thus, we feel that this methodology is generalizable to all cases. Although efforts were made to standardize grading, the subjective nature of Frisén grading in papilledema assessment may introduce some variability. All reading of IIHTT images were undertaken at designated reading centers with arbitration if there was disagreement. The normative data used came from several sources that are not as standardized as the photographs from the IIHTT, so interpretation of these data should be taken in this context. We did not use lumbar puncture results from individuals with a normal ICP, and we do not know whether there would be a similar correlation in this population. However, other groups have noted correlations in borderline ICP cases.^37^ Finally, the use of fellow eyes for validation may introduce some limitations, as vascular measurements between eyes are not truly independent. Although this approach is commonly used and remains the most practical option available, it should be recognized that it may slightly overestimate the robustness of the validation.

### Future Work

In this study, we have looked only at papilledema secondary to IIH. We hope to extend these findings in papilledema secondary to other causes, such as dural venous sinus thrombosis, intracranial arteriovenous shunts, or tumor. Other disorders that swell the ONH should alter the AutoMorph assessment of the retinal vasculature, perhaps with some disease-specific signature. For example, non-arteritic anterior ischemic optic neuropathy often has massive ONH swelling but no retrobulbar or intracranial hypertension. Any retinal vascular changes might reflect the local compartment syndrome in the ONH region. The utilization of technologies such as laser speckle flowgraphy may allow for visualization of dynamic changes in retinal and ONH blood flow, providing further insight into disease mechanisms.^44^ Further research is warranted to explore the long-term applicability of these biomarkers, their relationship with long-term visual function, and their role in guiding personalized treatment strategies. Additionally, future studies should further explore the relationship of vascular metrics to lumbar puncture opening pressures given the potential of this methodology as a non-invasive alternative to regular lumbar puncture.

## Conclusions

Automated retinal vessel analysis may enable earlier identification of effective treatment and improve clinical management of IIH. This study demonstrates that ACZ treatment in IIH patients leads to significant improvements in retinal vascular morphology, with a notable reduction in venule diameter, increase in arteriole diameter, and decrease in V:A ratio. These vascular changes closely correlate with improvements in papilledema severity and ONH volume, highlighting their potential role as non-invasive biomarkers for monitoring ICP and treatment efficacy. By incorporating retinal vascular metrics into clinical practice, clinicians may be able to better track disease progression, detect early treatment response, and ultimately improve visual outcomes for patients with IIH.

## Supplementary Material

Supplement 1
