# Supplementary material for: Automated Retinal Vascular Analysis Reveals Response to Acetazolamide in Idiopathic Intracranial Hypertension
Source: Transl Vis Sci Technol. 2025 Dec 4;14(12):9. doi: 10.1167/tvst.14.12.9 (PMC12697697; doi:10.1167/tvst.14.12.9)
Supplement: Supplement 1 [file tvst-14-12-9_s001.docx]

**Supplementary Material**

| **Variable** | **Timepoint** | **Group** | **Mean Change** | **SD Change** | **t-statistic** | **p-value** | **Cohen's d** | **p-value**  **(B-H correction)** |
| --- | --- | --- | --- | --- | --- | --- | --- | --- |
| **Frisén grade** | One month | ACZ | -0.686 | 0.883 | -5.551 | 0.000 | -0.777 | 0.000 |
|  |  | Placebo | -0.039 | 0.871 | -0.322 | 0.749 | -0.045 | 0.764 |
|  | Two months | ACZ | -0.861 | 1.125 | -4.592 | 0.000 | -0.765 | 0.000 |
|  |  | Placebo | -0.275 | 1.132 | -1.536 | 0.133 | -0.243 | 0.159 |
|  | Three months | ACZ | -1.100 | 1.057 | -6.580 | 0.000 | -1.040 | 0.000 |
|  |  | Placebo | -0.297 | 0.878 | -2.060 | 0.047 | -0.339 | 0.061 |
|  | Four months | ACZ | -1.267 | 1.100 | -4.461 | 0.001 | -1.152 | 0.002 |
|  |  | Placebo | -0.313 | 0.946 | -1.321 | 0.206 | -0.330 | 0.238 |
|  | Five months | ACZ | -1.000 | 1.173 | -3.516 | 0.003 | -0.853 | 0.007 |
|  |  | Placebo | -0.423 | 0.809 | -2.668 | 0.013 | -0.523 | 0.020 |
|  | Six months | ACZ | -1.118 | 1.008 | -6.465 | 0.000 | -1.109 | 0.000 |
|  |  | Placebo | -0.486 | 0.818 | -3.513 | 0.001 | -0.594 | 0.004 |
| **Venule width** | One month | ACZ | -4.587 | 11.037 | -2.968 | 0.005 | -0.416 | 0.010 |
|  |  | Placebo | 1.217 | 12.453 | 0.698 | 0.489 | 0.098 | 0.523 |
|  | Two months | ACZ | 1.297 | 10.449 | 0.745 | 0.461 | 0.124 | 0.499 |
|  |  | Placebo | 4.340 | 15.661 | 1.753 | 0.087 | 0.277 | 0.109 |
|  | Three months | ACZ | -4.356 | 14.077 | -1.957 | 0.058 | -0.309 | 0.074 |
|  |  | Placebo | -1.078 | 11.014 | -0.595 | 0.555 | -0.098 | 0.590 |
|  | Four months | ACZ | 0.570 | 11.578 | 0.191 | 0.852 | 0.049 | 0.852 |
|  |  | Placebo | 2.764 | 12.615 | 0.876 | 0.395 | 0.219 | 0.431 |
|  | Five months | ACZ | -1.539 | 19.659 | -0.323 | 0.751 | -0.078 | 0.764 |
|  |  | Placebo | 3.553 | 12.549 | 1.444 | 0.161 | 0.283 | 0.192 |
|  | Six months | ACZ | -6.281 | 17.000 | -2.155 | 0.039 | -0.370 | 0.052 |
|  |  | Placebo | -3.018 | 12.812 | -1.394 | 0.172 | -0.236 | 0.203 |
| **Arteriole width** | One month | ACZ | 1.369 | 7.443 | 1.314 | 0.195 | 0.184 | 0.227 |
|  |  | Placebo | 3.304 | 10.777 | 2.190 | 0.033 | 0.307 | 0.046 |
|  | Two months | ACZ | 7.878 | 9.400 | 5.028 | 0.000 | 0.838 | 0.000 |
|  |  | Placebo | 6.282 | 14.563 | 2.728 | 0.010 | 0.431 | 0.017 |
|  | Three months | ACZ | 5.821 | 8.271 | 4.451 | 0.000 | 0.704 | 0.000 |
|  |  | Placebo | 4.159 | 10.483 | 2.413 | 0.021 | 0.397 | 0.031 |
|  | Four months | ACZ | 8.602 | 11.180 | 2.980 | 0.010 | 0.769 | 0.017 |
|  |  | Placebo | 2.928 | 8.916 | 1.314 | 0.209 | 0.328 | 0.239 |
|  | Five months | ACZ | 7.270 | 9.586 | 3.127 | 0.007 | 0.758 | 0.013 |
|  |  | Placebo | 6.314 | 11.672 | 2.759 | 0.011 | 0.541 | 0.018 |
|  | Six months | ACZ | 4.354 | 8.366 | 3.034 | 0.005 | 0.520 | 0.010 |
|  |  | Placebo | 1.453 | 6.786 | 1.267 | 0.214 | 0.214 | 0.242 |
| **V : A ratio** | One month | ACZ | -0.058 | 0.077 | -5.436 | 0.000 | -0.761 | 0.000 |
|  |  | Placebo | -0.036 | 0.146 | -1.759 | 0.085 | -0.246 | 0.107 |
|  | Two months | ACZ | -0.076 | 0.096 | -4.709 | 0.000 | -0.785 | 0.000 |
|  |  | Placebo | -0.026 | 0.105 | -1.556 | 0.128 | -0.246 | 0.156 |
|  | Three months | ACZ | -0.097 | 0.099 | -6.239 | 0.000 | -0.986 | 0.000 |
|  |  | Placebo | -0.058 | 0.141 | -2.493 | 0.017 | -0.410 | 0.026 |
|  | Four months | ACZ | -0.092 | 0.107 | -3.334 | 0.005 | -0.861 | 0.010 |
|  |  | Placebo | -0.005 | 0.055 | -0.346 | 0.734 | -0.087 | 0.764 |
|  | Five months | ACZ | -0.086 | 0.123 | -2.879 | 0.011 | -0.698 | 0.018 |
|  |  | Placebo | -0.049 | 0.145 | -1.713 | 0.099 | -0.336 | 0.123 |
|  | Six months | ACZ | -0.095 | 0.118 | -4.694 | 0.000 | -0.805 | 0.000 |
|  |  | Placebo | -0.042 | 0.124 | -1.992 | 0.054 | -0.337 | 0.071 |

**Supplementary Table 1a.** Mean change from baseline in study eye Frisén grade, venule width, arteriole width and venule : arteriole (V:A) ratio across all timepoints.

| **Variable** | **Timepoint** | **Group** | **Mean Change** | **SD Change** | **t-statistic** | **p-value** | **Cohen's d** | | **p-value**  **(B-H correction)** |
| --- | --- | --- | --- | --- | --- | --- | --- | --- | --- |
| **Frisén grade** | One month | ACZ | -0.404 | 0.891 | -3.267 | 0.002 | -0.453 | 0.007 | |
|  |  | Placebo | -0.042 | 0.824 | -0.350 | 0.728 | -0.051 | 0.746 | |
|  | Two months | ACZ | -0.778 | 1.222 | -3.820 | 0.001 | -0.637 | 0.003 | |
|  |  | Placebo | -0.027 | 1.093 | -0.150 | 0.881 | -0.025 | 0.889 | |
|  | Three months | ACZ | -1.000 | 1.000 | -6.403 | 0.000 | -1.000 | 0.000 | |
|  |  | Placebo | -0.125 | 0.793 | -0.892 | 0.379 | -0.158 | 0.426 | |
|  | Four months | ACZ | -1.571 | 0.938 | -6.271 | 0.000 | -1.676 | 0.000 | |
|  |  | Placebo | -0.571 | 0.756 | -2.828 | 0.014 | -0.756 | 0.033 | |
|  | Five months | ACZ | -1.222 | 1.309 | -3.963 | 0.001 | -0.934 | 0.004 | |
|  |  | Placebo | -0.391 | 0.941 | -1.994 | 0.059 | -0.416 | 0.090 | |
|  | Six months | ACZ | -1.143 | 1.033 | -6.545 | 0.000 | -1.106 | 0.000 | |
|  |  | Placebo | -0.344 | 0.827 | -2.350 | 0.025 | -0.415 | 0.049 | |
| **Venule width** | One month | ACZ | -3.494 | 13.849 | -1.819 | 0.075 | -0.252 | 0.108 | |
|  |  | Placebo | 2.525 | 11.541 | 1.516 | 0.136 | 0.219 | 0.176 | |
|  | Two months | ACZ | -4.058 | 14.981 | -1.625 | 0.113 | -0.271 | 0.150 | |
|  |  | Placebo | 9.529 | 16.826 | 3.445 | 0.001 | 0.566 | 0.006 | |
|  | Three months | ACZ | -6.246 | 16.152 | -2.476 | 0.018 | -0.387 | 0.036 | |
|  |  | Placebo | 1.242 | 9.326 | 0.753 | 0.457 | 0.133 | 0.498 | |
|  | Four months | ACZ | 1.919 | 8.435 | 0.851 | 0.410 | 0.228 | 0.456 | |
|  |  | Placebo | 5.322 | 13.639 | 1.460 | 0.168 | 0.390 | 0.212 | |
|  | Five months | ACZ | -8.394 | 24.523 | -1.452 | 0.165 | -0.342 | 0.210 | |
|  |  | Placebo | -0.217 | 19.397 | -0.054 | 0.958 | -0.011 | 0.958 | |
|  | Six months | ACZ | -10.068 | 18.429 | -3.232 | 0.003 | -0.546 | 0.009 | |
|  |  | Placebo | 2.243 | 11.989 | 1.058 | 0.298 | 0.187 | 0.351 | |
| **Arteriole width** | One month | ACZ | 3.259 | 11.214 | 2.096 | 0.041 | 0.291 | 0.072 | |
|  |  | Placebo | 2.031 | 8.897 | 1.582 | 0.120 | 0.228 | 0.157 | |
|  | Two months | ACZ | 5.964 | 12.827 | 2.790 | 0.008 | 0.465 | 0.021 | |
|  |  | Placebo | 8.840 | 14.771 | 3.640 | 0.001 | 0.598 | 0.004 | |
|  | Three months | ACZ | 5.507 | 11.231 | 3.140 | 0.003 | 0.490 | 0.010 | |
|  |  | Placebo | 2.899 | 9.421 | 1.740 | 0.092 | 0.308 | 0.129 | |
|  | Four months | ACZ | 10.060 | 9.379 | 4.013 | 0.001 | 1.073 | 0.006 | |
|  |  | Placebo | 7.471 | 8.145 | 3.432 | 0.004 | 0.917 | 0.013 | |
|  | Five months | ACZ | 7.361 | 17.346 | 1.800 | 0.090 | 0.424 | 0.128 | |
|  |  | Placebo | 8.548 | 19.434 | 2.110 | 0.047 | 0.440 | 0.080 | |
|  | Six months | ACZ | 4.484 | 13.401 | 1.979 | 0.056 | 0.335 | 0.088 | |
|  |  | Placebo | 2.636 | 8.060 | 1.850 | 0.074 | 0.327 | 0.108 | |
| **V : A ratio** | One month | ACZ | -0.074 | 0.117 | -4.502 | 0.000 | -0.630 | 0.000 | |
|  |  | Placebo | 0.011 | 0.125 | 0.621 | 0.537 | 0.090 | 0.576 | |
|  | Two months | ACZ | -0.106 | 0.128 | -4.961 | 0.000 | -0.827 | 0.000 | |
|  |  | Placebo | -0.008 | 0.103 | -0.480 | 0.634 | -0.079 | 0.668 | |
|  | Three months | ACZ | -0.114 | 0.121 | -5.983 | 0.000 | -0.946 | 0.000 | |
|  |  | Placebo | -0.019 | 0.096 | -1.112 | 0.275 | -0.197 | 0.330 | |
|  | Four months | ACZ | -0.094 | 0.107 | -3.270 | 0.006 | -0.874 | 0.016 | |
|  |  | Placebo | -0.033 | 0.084 | -1.452 | 0.170 | -0.388 | 0.213 | |
|  | Five months | ACZ | -0.135 | 0.100 | -5.710 | 0.000 | -1.346 | 0.000 | |
|  |  | Placebo | -0.089 | 0.101 | -4.192 | 0.000 | -0.874 | 0.002 | |
|  | Six months | ACZ | -0.140 | 0.134 | -6.105 | 0.000 | -1.047 | 0.000 | |
|  |  | Placebo | -0.010 | 0.139 | -0.412 | 0.683 | -0.073 | 0.713 | |

**Supplementary Table 1b.** Mean change from baseline in fellow eye Frisén grade, venule width, arteriole width and venule : arteriole (V:A) ratio across all timepoints.

| **Timepoint** | **ACZ or Placebo** | **Dropped Photos** | **Unique Eyes** | **Unique Patients** | **Mean Frisén Grade** |
| --- | --- | --- | --- | --- | --- |
| Baseline | ACZ | 58 | 21 | 15 | 3.47 |
|  | Placebo | 22 | 12 | 10 | 3.86 |
| One month | ACZ | 25 | 10 | 8 | 2.88 |
|  | Placebo | 43 | 11 | 9 | 3.7 |
| Two months | ACZ | 3 | 3 | 2 | 2.33 |
|  | Placebo | 15 | 3 | 2 | 4 |
| Three months | ACZ | 10 | 4 | 4 | 2.5 |
|  | Placebo | 5 | 3 | 2 | 1.6 |
| Five months | ACZ | 1 | 1 | 1 | 2 |
|  | Placebo | 3 | 2 | 2 | 3.33 |
| Six months | ACZ | 8 | 4 | 3 | 1 |
|  | Placebo | 6 | 6 | 5 | 2.5 |

**Supplementary Table 2.** Number of automatically excluded (low-quality) optic nerve head images by treatment arm and visit, showing dropped photos, unique eyes, unique patients, and mean Frisén grade. Most exclusions occurred at the baseline visit (58 ACZ, 22 Placebo). The mean Frisén grade among excluded photos was 2.4 in ACZ and 3.2 in Placebo, suggesting that image rejection was not strongly biased toward higher-grade swelling. A manual check of a subset of excluded images revealed that excluded images tended to be poorly focused, had poor lighting, or imaging artefacts.

| **Metric** | **Standardized Estimate** | **CI lower** | **CI upper** | **p-value** | **no. of subjects** |
| --- | --- | --- | --- | --- | --- |
| Frisén Grade | -0.104 | -0.151 | -0.057 | <0.001 | 151 |
| V:A Width Ratio | -0.081 | -0.132 | -0.029 | 0.001 | 151 |
| V:A Vessel Density Ratio | -0.055 | -0.109 | -0.001 | 0.046 | 151 |
| Venule Average Width | -0.014 | -0.042 | 0.013 | 0.315 | 151 |
| Venule Vessel Density | 0.033 | -0.016 | 0.084 | 0.189 | 151 |
| Arteriole Average Width | 0.026 | -0.002 | 0.054 | 0.076 | 151 |
| Arteriole Vessel Density | 0.069 | 0.019 | 0.118 | 0.006 | 151 |

**Supplementary Table 3**. **Standardized Over-Time Treatment Effects (ACZ - Placebo) from Unified Mixed-Effects Models.** Standardized effect estimates from linear mixed-effects models evaluating the interaction between time and treatment across all study visits. Each value represents the difference in rate of change between acetazolamide (ACZ) and placebo, expressed in standardized (z-score) units per month. Positive estimates indicate greater increase (or lesser decline) with ACZ relative to placebo, and negative estimates indicate greater reduction with ACZ.


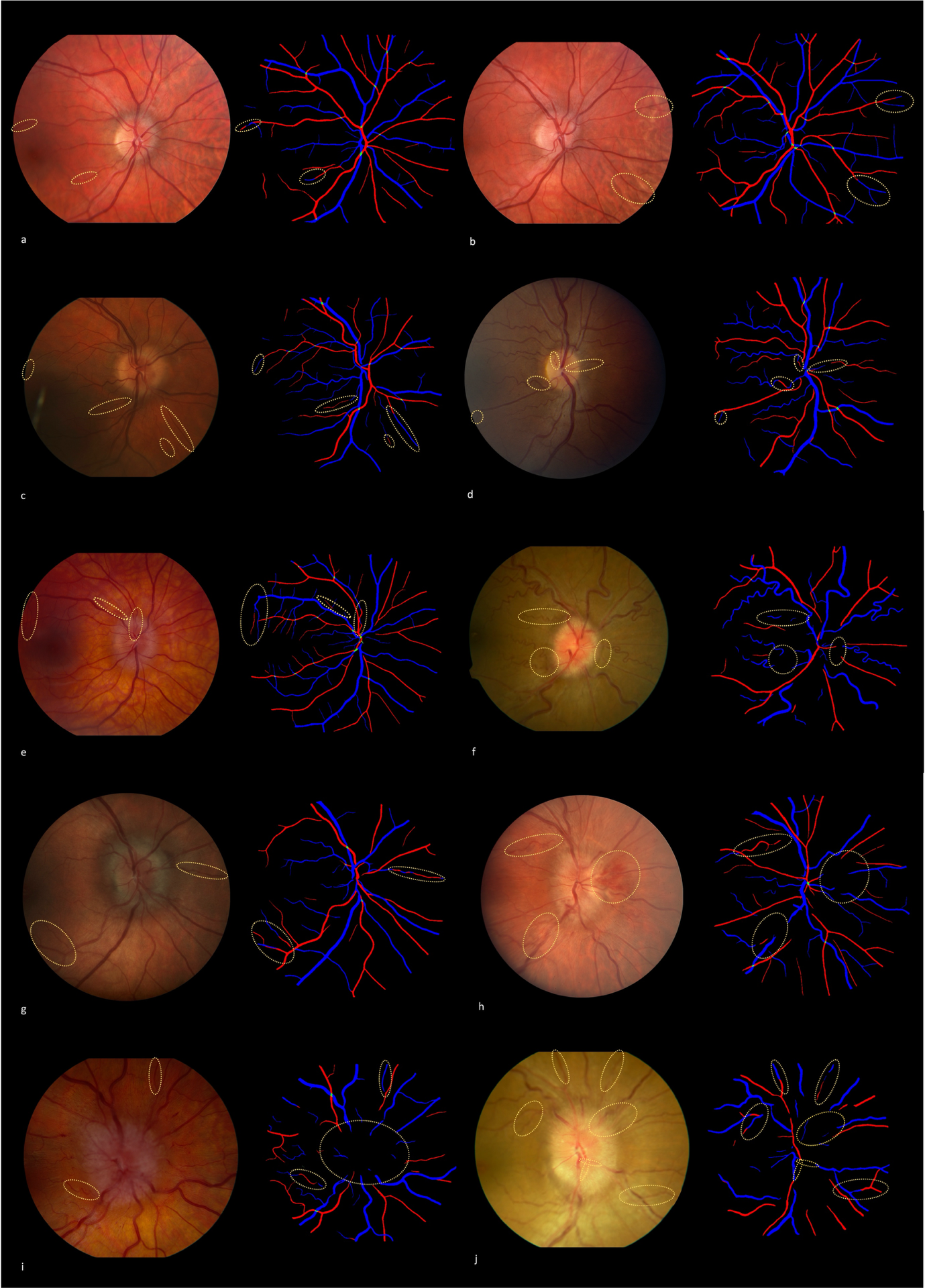


**Supplementary Figure 1.** Examples of vessel segmentation failure and misclassification for Frisén grade 1 (a, b), grade 2 (c, d), grade 3 (e, f), grade 4 (g, h), and grade 5 (i, j). Fundus photographs and corresponding venule/arteriole overlays are shown, with ellipses indicating areas of segmentation failure or confusion. Misclassification of vessels tended to occur more peripherally or in regions containing small, fine vessels that were out of focus (a-g). Increased segmentation failures were noted in cases of very severe swelling or hemorrhage (h-j). First-order vessels were rarely misclassified.


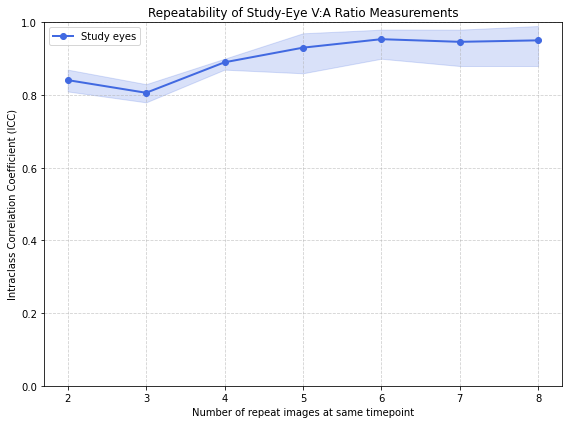


**Supplemental Figure 2.** Repeatability of Study-Eye V:A Ratio Measurements. Intraclass correlation coefficients (ICCs) for V:A width ratio measurements from study eyes increased with additional repeated fundus photographs per timepoint were included in the analysis, indicating progressively higher measurement reliability. Shaded regions denote 95% confidence intervals.
